# Racial Differences in Genomic Profiles of Breast Cancer

**DOI:** 10.1001/jamanetworkopen.2022.0573

**Published:** 2022-03-01

**Authors:** Neha Goel, Daniel Y. Kim, Jimmy A. Guo, Daniel Zhao, Brandon A. Mahal, Mohammed Alshalalfa

**Affiliations:** 1 Sylvester Comprehensive Cancer Center, University of Miami Miller School of Medicine, Miami, Florida; 2 Harvard Medical School, Boston, Massachusetts; 3 Broad Institute of MIT and Harvard, Cambridge, Massachusetts; 4 Department of Human Genetics, University of California, Los Angeles, Los Angeles

## Abstract

This genetic association study examines the tumor genomic profiles by race in a large, diverse patient cohort using next-generation sequencing (NGS) data in the American Association for Cancer Research Project Genomics Evidence Neoplasia Information Exchange.

## Introduction

Racial disparities in breast cancer outcomes remain a persistent challenge.^1^ Earlier onset, advanced stage at diagnosis, aggressive tumor subtype (eg, triple-negative breast cancer), and shorter survival are characteristic features of breast cancer in Black patients compared with their White counterparts, denoting one of the most significant examples of racial or ethnic inequality in oncology.^2,3^ The etiology of these disparities spans socioeconomic, environmental, and genomic factors. Recent advancements in precision oncology contribute to these disparities by underrepresenting Black patients and Asian patients, limiting the discovery of variations and potentially targetable genes in diverse populations.^4^ To bridge this critical gap, we examined tumor genomic profiles by race in a large, diverse patient cohort.

## Methods

Because of the use of publicly available, deidentified data, this study was exempt from institutional review board approval at the University of Miami, and informed consent was waived. This study followed the Strengthening the Reporting of Observational Studies in Epidemiology (STROBE) reporting guideline.

A major goal of this genetic association study was to evaluate the racial differences in genomic profiles of breast cancer in a large national cohort since the majority of studies in precision oncology only focus on White women. Race and ethnicity were self-reported and included Asian individuals, Black individuals, Native American individuals, Pacific Islander individuals, and White individuals. There were 9 Native American patients and 2 Pacific Islander patients. Thus, we decided to move forward using the data from Asian patients, Black patients, and White patients because these groups had enough data to complete a well-powered analysis. In addition, there are Hispanic and non-Hispanic annotations; however, a subgroup analysis between Hispanic White patients and non-Hispanic White patients would have led to unequal groups with small numbers and limited power. Therefore, Hispanic White patients and non-Hispanic White patients were grouped together.

Patients with breast cancer treated at Memorial Sloan Kettering and Dana Farber Cancer Institute from 2014 to 2020 with complete clinical and next-generation sequencing (NGS) data in the American Association for Cancer Research Project Genomics Evidence Neoplasia Information Exchange (GENIE) were included.^5^ Variation profiles of 642 genes were examined by self-reported race (Asian patients, Black patients, White patients) and tumor location (primary or metastatic). Benjamini–Hochberg method was used to control for the false discovery rate (eAppendix in the Supplement).

The software package R, version 4.0.3 (R Project for Statistical Computing) was used for statistical calculations. The *z* test was used to calculate *P* values, *P* < .05 was considered statistical significance, and tests were 2-sided.

## Results

**Figure 1.**
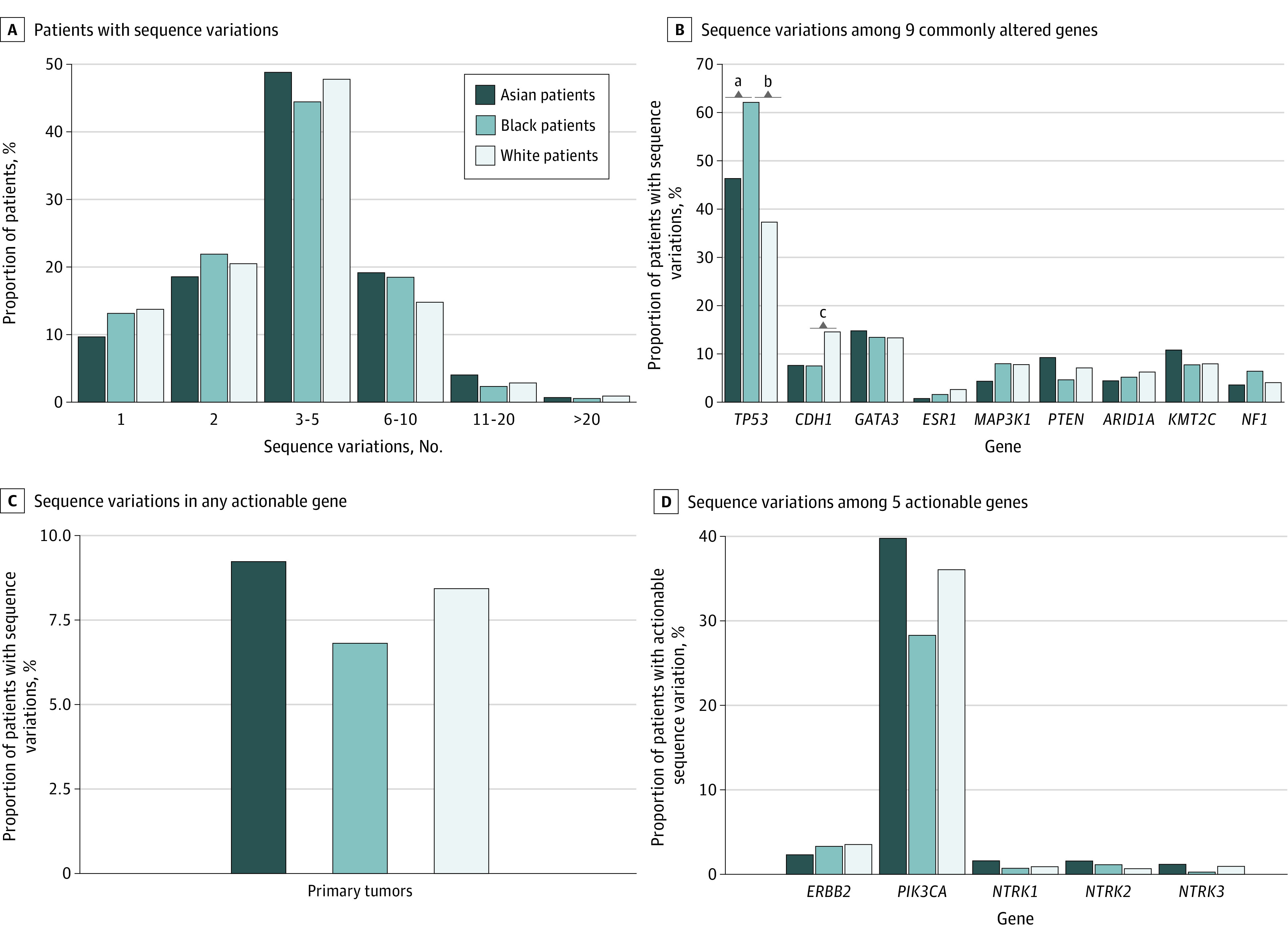
Tumor Variation Profiles of 3557 Patients With Primary Breast Cancer Panel A, percentage of patients with 1, 2, 3-5, 6-10, 11-20, or >20 variations among those who were sequenced on the Memorial Sloan Kettering-468 sequencing panel. Panel B, variation frequencies for 9 genes that were commonly altered in the study. Panel C, percentage of patients with variations in any actionable gene. Panel D, variation frequencies for 5 actionable genes. Genes with actionable variations include *ERBB2*, *NTRK1/2/3*, and *PIK3CA*.

**Figure 2.**
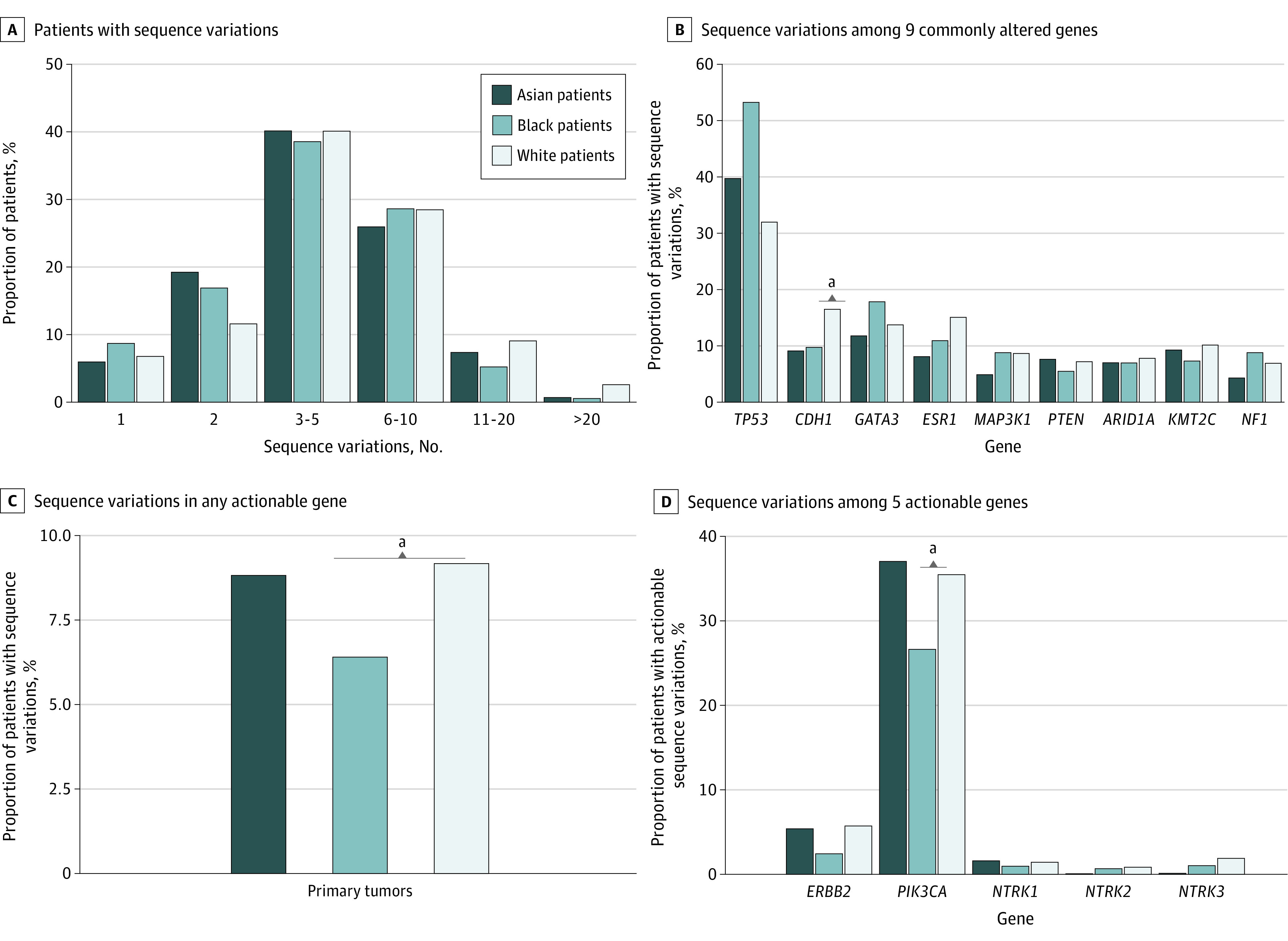
Tumor Variation Profiles of 3095 Patients With Metastatic Breast Cancer Panel A shows the percentage of patients with 1, 2, 3 to 5, 6 to 10, 11 to 20, or more than 20 variation among those who were sequenced on the Memorial Sloan Kettering-468 sequencing panel. Panel B shows the variation frequencies for 9 genes that were commonly altered in the study. Panel C shows the percentage of patients with variations in any actionable gene. Panel D shows the variation frequencies for 5 actionable genes. Genes with actionable variations include *ERBB2*, *NTRK1/2/3*, and *PIK3CA*.

Among 6652 patients with a median (IQR) age of 56 (17) years, 3.5% had primary disease sequenced (7.1% Asian patients, 9.2% Black patients, 83.8% White patients), and 46.5% had metastatic disease sequenced (6.0% Asian patients, 8.9% Black patients, 85.1% White patients). The variation profiles for primary and metastatic breast cancer by race are shown in Figure 1 and Figure 2, respectively.

Among patients with NGS on primary breast cancer, *TP53* variations occurred more often in Black patients than White patients (62.0% vs 37.2%; unadjusted between-group difference [referred to as difference throughout], 24.8%; 95% CI, 19.0-30.5; *P* < .001) and Asian patients (62.0% vs 46.2%; difference, 15.8%; 95% CI, 7.3-24.2; *P* < .008) (Figure 1). *CDH1* mutations occurred more frequently in White patients compared with Black patients (14.5% vs 7.7%; difference, 6.8%; 95% CI, 3.5-10.1; *P* = .02) (Figure 1).

Among patients with NGS on metastatic breast cancer, *CDH1* variations occurred more often in White patients than Black patients (16.6% vs 9.9%; difference, 6.7%; 95% CI, 2.7 to 10.7; *P* = .04) (Figure 2). Genes with actionable variations occurred less often in Black patients than in White patients (6.4% vs 9.1%; difference, −2.7; 95% CI, −4.1 to −1.3; *P* < .05) (Figure 2). Specifically, *PIK3CA* targetable actionable variations occurred more frequently in White patients compared with Black patients (35.5% vs 26.6%; difference, 8.9%; 95% CI, 3.1 to 14.6; *P* = .04) (Figure 2).

## Discussion

In this study, we identified that Black patients with metastatic breast cancer were less likely than White patients or Asian patients to have actionable genetic variations, specifically in *PIK3CA*. These findings are clinically relevant as results of a randomized, phase 3 trial comparing alpelisib (*PIK3CA* inhibitor) plus fulvestrant or placebo plus fulvestrant in hormone receptor-positive, human epidermal growth factor receptor 2-negative metastatic breast cancer with relapse or progression during or after endocrine therapy showed a significantly prolonged progression-free survival and a greater response with alpelisib plus fulvestrant than with placebo plus fulvestrant.^6^

To our knowledge, this is the largest study of racial differences in genomic profiles. Limitations include the lack of breast cancer subtype data in GENIE and potential institutional differences in NGS eligibility. However, although we are unable to assess genomic profiles by subtype, our focus on racial differences in mutations of actionable genes still highlights the importance of increasing minority enrollment in clinical trials and accessibility to precision oncology. This enables us to further explore breast cancer outcomes in the context of race-sensitive treatment paradigms and inform the novel discovery of new targetable and actionable genes in minority populations. The clinical implications of such data could help narrow the persistent racial gap in breast cancer outcomes.

## Notes

Supplement.
eMethods.

eReferences.
