# Supplementary material for: Racial Differences in Genomic Profiles of Breast Cancer
Source: JAMA Netw Open. 2022 Mar 1;5(3):e220573. doi: 10.1001/jamanetworkopen.2022.0573 (PMC8889463; doi:10.1001/jamanetworkopen.2022.0573)
Supplement: Supplement. — eMethods. eReferences. [file jamanetwopen-e220573-s001.pdf]

Supplemental Online Content

Goel N, Kim DY, Guo JA, Zhao D, Mahal BA, Alshalalfa M. Racial differences in genomic profiles of breast cancer. *JAMA Netw Open*. 2022;5(3):e220573. doi:10.1001/jamanetworkopen.2022.0573

eMethods.
eReferences.

This supplemental material has been provided by the authors to give readers additional information about their work.

© 2022 Goel N et al. *JAMA Network Open.*

eMethods.

Patient Cohort

Next generation sequencing (NGS) tumor genomic profiles (somatic non-synonymous genomic alteration data) of patients treated for primary or metastatic breast cancer were identified from the American Association for Cancer Research (AACR) Project Genomics Evidence Neoplasia Information Exchange (GENIE) 9.0-registry (released February 2021).[1,2] The GENIE v9.0-registry contains genomic data that is obtained during the course of routine practice at multiple participating institutions. The AACR Project GENIE is a multiphase, multi-year, data-sharing project that captures genomic data for cancer patients across a multiple international institutions. As described in the AACR Project GENIE Data Guide (https://www.aacr.org/wp-content/uploads/2021/02/GENIE_9.0-public_data_guide.pdf), "the database currently contains CLIA-/ISO-certified genomic data obtained during the course of routine practice at multiple international institutions (Table 1) and will continue to grow as more patients are treated at additional participating centers."

The majority of breast cancer cases (63%) in the GENIE v9.0-registry are captured by Memorial Sloan Kettering Cancer Center (MSK)-IMPACT and Dana-Farber Cancer Institute (DFCI)-Oncopanel NGS initiatives. MSK and DFCI were the only institutions with race demographic data that also captured >6% of breast cancer patients in the registry. Therefore, MSK and DFCI samples were used for cohort selection. Data were downloaded from cBioPortal (v3.6.12) GENIE cohort v9.0.

© 2022 Goel N et al. *JAMA Network Open.*

In our cohort, 1.2% of primary tumors were male and 98.8% of primary tumors were females. Similarly, 0.7% of metastatic tumors were male and 99.3% of metastatic tumors were females. The average age of Asian, Black, and White individuals with primary tumors was 48, 55, and 56 years, respectively. Likewise, for metastatic tumors, the average age of Asian, Black, and White individuals was 52, 55, and 58, respectively.

Genomic Sequencing Statistical Methods

MSK and DFCI each contributed NGS data from 3 different panels (with increasing coverage) to the GENIE 9.0-registry (MSK 341, MSK 410, MSK 468, DFCI 1, DFCI 2, DFCI 3); specifics about the DFCI and MSK NGS assays are described below.[1,2] Using these NGS panels, we analyzed 642 unique genes, including all actionable targets (i.e., genes that are targeted by therapies and that provide information about the disease); Mutational frequencies of relevant genes were calculated and compared across race using a two-proportion z-test with Yates' continuity correction. To account for panel differences, the denominator used to calculate mutational frequency for each gene included the number of patients who received sequencing for each specific gene, rather than the total number of patients sequenced. The Benjamini-Hochberg method was used to correct for multiple testing, with $P<0.05$ considered significant.

Next Generation Sequencing Assays

Specifics about genomic profiling at each center are provided below, as copied from the AACR Project GENIE Data Guide: (https://www.aacr.org/wp-content/uploads/2021/02/GENIE_9.0-public_data_guide.pdf).

© 2022 Goel N et al. *JAMA Network Open.*

Dana-Farber Cancer Institute (DFCI)

“DFCI uses a custom, hybridization-based capture panel (OncoPanel) to detect single nucleotide variants, small indels, copy number alterations, and structural variants from tumor-only sequencing data. Three (3) versions of the panel have been submitted to GENIE: version 1 containing 275 genes, version 2 containing 300 genes, version 3 containing 447 genes. Specimens are reviewed by a pathologist to ensure tumor cellularity of at least 20%. Tumors are sequenced to an average unique depth of coverage of approximately 200x for version 1 and 350x for version 2. Reads are aligned using BWA, flagged for duplicate read pairs using Picard Tools, and locally realigned using GATK. Sequence mutations are called using MuTect for SNVs and GATK SomaticIndelDetector for small indels. Putative germline variants are filtered out using a panel of historical normals or if present in ESP at a frequency >= .1%, unless the variant is also present in COSMIC. Copy number alterations are called using a custom pipeline and reported for fold-change >1. Structural rearrangements are called using BreaKmer. Testing is performed for all patients across all solid tumor types. Version 3 includes the exonic regions of 447 genes and 191 intronic regions across 60 genes targeted for rearrangement detection. 52 genes present in previous versions were retired in the v3 test.”

Memorial Sloan Kettering Cancer Center (MSK)

“MSK uses a custom, hybridization-based capture panel (MSK-IMPACT) to detect single nucleotide variants, small indels, copy number alterations, and structural variants from matched tumor-normal sequence data. Three (3) versions of the panel have been submitted to GENIE: version 1 containing 341 genes, version 2 containing 410 genes, version 3 containing 468 genes. Specimens are reviewed by a pathologist to ensure tumor cellularity of at least 10%. Tumors are

© 2022 Goel N et al. *JAMA Network Open.*

sequenced to an average unique depth of coverage of approximately 750X. Reads are aligned using BWA, flagged for duplicate read pairs using GATK, and locally realigned using ABRA. Sequence mutations are called using MuTect, VarDict, and Somatic indel detector, and reported for >5% allele frequency (novel variants) or >2% allele frequency (recurrent hotspots). Copy number alterations are called using a custom pipeline and reported for fold-change >2. Structural rearrangements are called using Delly. All somatic mutations are reported without regard to biological function. Testing is performed for patients with advanced metastatic cancer across all solid tumor types."

© 2022 Goel N et al. *JAMA Network Open.*

eReferences.

1. Zehir A, Benayed R, Shah RH, et al. Mutational landscape of metastatic cancer revealed from prospective clinical sequencing of 10,000 patients. *Nat Med.* 2017;23(6):703-713.

2. AACR Project GENIE: Powering Precision Medicine through an International Consortium. *Cancer Discov.* 2017;7(8):818-831.

© 2022 Goel N et al. *JAMA Network Open.*
